# Proceedings from an Indigenous Women’s Health Workshop: Use of a Co-Creation Process to Build Cross-Disciplinary Relationships and Support Creation of an Indigenous Women’s Health Priority Agenda

**DOI:** 10.3390/ijerph22030390

**Published:** 2025-03-07

**Authors:** Chevelle M. A. Davis, Reni Soon, Kaitlyn Aoki, Kelli Begay, Denise Charron-Prochownik, Rebecca Dendy, Jennifer Elia, Heather Garrow, Kapuaola Gellert, Luciana E. Hebert, Mary Hoskin, Megan Kiyomi Inada, Bliss Kaneshiro, Ka’ōnohi Lapilo, Kelly R. Moore, Sharon Kaiulani Odom, Diane Paloma, Mei Linn Park, Lisa Scarton, Susan Sereika, Marjorie K. L. M. Mau, Sarah A. Stotz

**Affiliations:** 1 Robert Wood Johnson Foundation Health Policy Research Scholar Alumni, Hawai‘i Children’s Action Network, Honolulu, HI 96822, USA; cdavis@hawaii-can.org; 2 Department of Obstetrics, Gynecology, and Women’s Health, John A. Burns School of Medicine, Queens Health Systems, Honolulu, HI 96813, USA; rsoon@hawaii.edu (R.S.); blissk@hawaii.edu (B.K.); 3 Department of Native Hawaiian Health, John A. Burns School of Medicine, Honolulu, HI 96813, USA; aokikait@hawaii.edu (K.A.); lapilio2@hawaii.edu (K.L.); 4 Independent Researcher, Edmond, OK 73012, USA; kelli@mavencollectiveconsulting.com; 5 School of Nursing, University of Pittsburgh, Pittsburgh, PA 15213, USA; dcpro@pitt.edu (D.C.-P.); ssereika@pitt.edu (S.S.); 6 College of Medicine, University of Arizona, Tucson, AZ 85724, USA; rdendy@arizona.edu; 7 Early Childhood Action Strategy for Hawai’i Maternal and Infant Health Collaborative, Honolulu, HI 96823, USA; jennifer@ecashawaii.org; 8 Saint Regis Mohawk Tribe, Akwesasne, NY 13655, USA; heather.garrow@srmt-nsn.gov; 9 John A. Burns School of Medicine, University of Hawai‘i at Mānoa, Honolulu, HI 96813, USA; sgellert@hawaii.edu (K.G.); mmau@hawaii.edu (M.K.L.M.M.); 10 Institute for Research and Education to Advance Community Health, Washington State University, Seattle, WA 98101, USA; luciana.hebert@wsu.edu; 11 National Institutes of Diabetes, Digestive, and Kidney Disease at Phoenix Diabetes Epidemiology and Clinical Research Section, Phoenix, AZ 85016, USA; mhoskin@mail.nih.gov; 12 Kōkua Kalihi Valley, Honolulu, HI 96819, USA; minada@kkv.net; 13 Center for American Indian and Alaska Native Health, University of Colorado Anschutz Medical Campus, Aurora, CO 80045, USA; kelly.moore@cuanschutz.edu; 14 Hawaii Good Food Alliance, Honolulu, HI 96822, USA; kaiu@hawaiigoodfoodalliance.org; 15 Hawai’i Dental Service, Honolulu, HI 96813, USA; diane.paloma@gmail.com; 16 Department of Native Hawaiian Health, University of Hawai’i, Honolulu, HI 96813, USA; meip@hawaii.edu; 17 School of Nursing, University of Florida, Gainesville, FL 32603, USA; lscarton@ufl.edu; 18 Department of Food Science and Human Nutrition, Colorado State University, Fort Collins, CO 80526, USA

**Keywords:** Indigenous women’s health, intersectionality, consensus building, Indigenous strength-based values

## Abstract

Indigenous women experience disproportionately higher rates of adverse health outcomes. Few studies have explored the root of these problems or defined health and wellness from the perspectives of Indigenous women. Our objective was to elicit views on Indigenous women’s health from women who are Indigenous and/or have experience working with Indigenous communities across Turtle Island and Hawai‘i (e.g., United States). Informed by intersectionality as a social critical theory, we convened a workshop to engage in a co-creative consensus-building and expert decision process using design thinking. The two-day workshop embraced Indigenous values of land, sacred spaces, genealogy, family, rituals, and culture. Participants included United States-based Native and Indigenous women (n = 16) and allies (n = 7). Participants focused on answering key questions such as “What are priority areas for Indigenous women’s health”? and “What are the key facilitators and barriers to improving Indigenous women’s health”? Co-created priority lists for each of these topics were generated. Participants overwhelmingly reported satisfaction with the workshop process and emphasis on a strength-based, culturally driven approach to share their stories, which contextualized the ideas, concerns, and priorities of Indigenous women who self-reflected on their own health and wellness. Creating culturally safe spaces for Indigenous people to reflect on their own hopes for the future relates to the theme by describing a process to bridge traditional healing with modern-day practices to build pilina.

## 1. Introduction

Indigenous women experience disproportionately higher rates of adverse health outcomes in such areas as heart health [1], cancer [2], and maternal morbidity and mortality [3]. This occurs because of a combination of many factors influenced by the unique roles women and two-spirit people hold as cultural and familial caretakers, the impacts of historical trauma on maternal and reproductive health, and the social drivers of health such as limited healthcare access, built environment (e.g., unsafe living conditions), economic stability (e.g., poverty) [4,5]. Though the literature recognizes the ‘upstream’ causes of these disparities [5], including colonization [6], anti-Indigenous racism [7,8], forced removal from Native homelands [9], and intentional destruction of healthful cultural practices [10], few studies have explored the roots of these problems or defined health and wellness from the perspective of Indigenous women. Findings of the qualitative aim of our team’s recently completed food insecurity and gestational diabetes-focused project indicate that Indigenous women and experts in the areas of Indigenous women’s health, culture, and social drivers of health recommend de-siloing efforts to improve Indigenous women’s health [11,12] by working across sectors (e.g., healthcare, social services, housing, environmental, etc.) and across health care disciplines (e.g., mental health and OB/GYN). Further, rather than focusing on disease-specific risk reduction or trying to adapt risk reduction methods for other populations to serve Indigenous people’s needs, efforts to improve Indigenous women’s health should be disease-agnostic and center on Indigenous strengths, values, ways of knowing, and holistic health [11,12].

To further engage the priority audience and community-based participatory research principles [13,14,15], our team convened an Indigenous Women’s Health Workshop. We employed a design-thinking and data-driven approach to engage a multidisciplinary team of women with clinical, research, and lived expertise related to this topic. Our objective for this workshop was to create a space for nurturing and eliciting intentional dialogue on interdisciplinary views on Indigenous women’s health from women who are Indigenous and/or have experience working with Indigenous communities. This manuscript presents the methods and findings from this workshop.

## 2. Materials and Methods

Informed by intersectionality as a social critical theory [16,17] and community-based participatory research [13,14], we engaged in a co-creative consensus building, design thinking, [18,19] and expert decision process [18] with an invited group of Indigenous women who work in various aspects of Indigenous women’s health. Participants (n = 23) included researchers, professors, physicians, nurses, nurse practitioners, social workers, registered dietitian nutritionists, traditional healers, epidemiologists, dentists, social scientists, and graduate students—most of whom also identified as mothers, daughters, grandmothers, aunts, sisters, and caretakers. Of these participants, 16 identified as American Indian or Native Hawaiian, and 7 identified as allies who strive to learn from and advocate for Native and Indigenous rights, health, and wellbeing.

We carefully considered how to create a safe space that was grounded in Indigenous values. Given this workshop took place in Hawai`i, the local collaborators, most of whom were of Native Hawaiian descent, recommended locally owned and operated vendors, caterers, and food suppliers as well as a Native-owned and operated venue, called the Waiwai Collective [20]. Additionally, the two-day workshop was intentionally conversation-based talk-story circles (e.g., casual, unstructured conversation inclusive of storytelling), and culturally appropriate protocols were led by Native Hawaiian participants [e.g., land acknowledgment, *oli* (chant), *mele* (song), and *pule* (prayer).]

Within the group, we also considered equity and power dynamics and employed strategies to redistribute power and ensure equity among the participants. For example, some participants currently work in positions that are not salaried, and, for these participants, we provided an hourly paid stipend for their time. Additionally, all meals and parking fees were covered for all participants. We formed small group breakout sessions to accommodate inherent power dynamics (e.g., Elders sharing space with students and/or junior researchers and clinicians). This allowed members who were uncomfortable contributing to the larger group to engage more fully in the discussion. The composition of the small groups also changed throughout the workshop to allow for group variability. Talking circles ensured that all participants had the time and space to express their thoughts and have their voices heard.

Using a co-creation consensus-building (e.g., the whole group working together without a designated ‘lead’ to the conversation) and expert discovery process [18] and principles from a traditional conversation-based dialogue in Hawai`i called ‘talk story,’ we employed small group breakout sessions to first determine the questions to focus on and the precise methods to be used to answer those questions. After each small group breakout discussion and large group share back, we used a designed thinking approach to individually and iteratively vote on priority areas concerning Indigenous women’s health, facilitators to promote Indigenous women’s health, and intervention strategies that arose, and we co-created key goals to address these priority areas [18,19]. After developing goals, the group ranked them based on impact and feasibility. All participants voted on the co-created priority areas in Indigenous women’s health with a democratic voting system, which allowed every participant to have equal power in determining the highest-ranked priority areas.

## 3. Results

**Figure 1:**
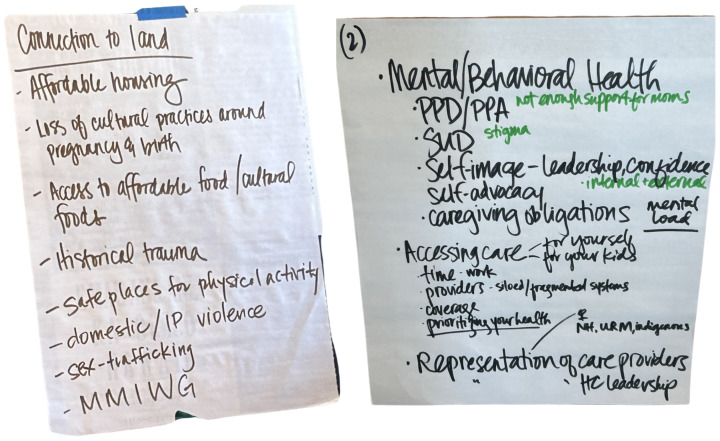
Examples of small groups’ brainstorming activities.

Guided by consensus of the group, the first breakout session on Day 1 focused on generating ideas on key health priorities for Indigenous women and facilitators/challenges to addressing these key priorities. The two specific prompts were: (1) What are key health priorities for Indigenous women’s health? and (2) What are key challenges and facilitators to supporting Indigenous women’s health? Three small groups of 4–6 people spent ~2 h discussing their responses to this query. Each group selected a note-taker who used large flipchart paper to memorialize key topics discussed in the small group, and another small group member to lead the share-back presentation. Each group then provided a share-back summary of their breakout group discussion, and the workshop organizers created a list of areas discussed during each of the three groups’ share-back summary presentations. Examples of small groups’ brainstorming documents used for share-back summary presentations can be found in Figure 1.

**Figure 2:**
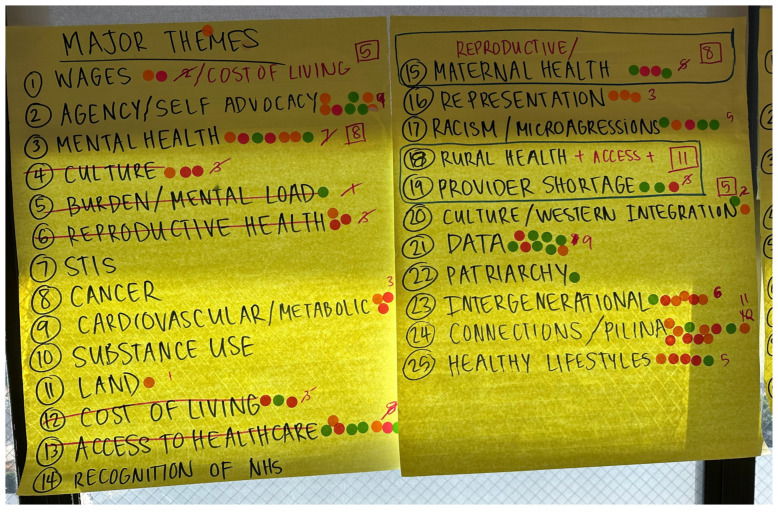
Voting document used after breakout sessions on Day 1 to collectively construct key themes.

Next, each workshop participant was given five stickers to “vote” on their priority topics. This voting process occurred during an informal break in the workshop for the process to be more comfortable and anonymous, and for individuals to feel free to vote as they wished, and to take their time to allocate their votes. Finally, collectively, the entire group then rearranged and organized the voted-upon key priority areas and challenges where redundancy was evident. For example, “wages” and “cost of living” were noted as similar challenges and were grouped together as one topic. An example of the voting document can be found in Figure 2.

In response to the first-day breakout group session question “What are key health priorities for Indigenous women, and the salient facilitators and challenges to actualizing these health priorities”? the final list generated during the workshop can be found in Table 1. The lists are in alphabetical order by topic, as the group preferred not to use a hierarchical approach in this phase of the process.

Day 2 of the workshop included a large group sharing session focused on what each participant was doing presently and what each hoped to do concerning Indigenous women’s health in the future. Participants shared their stories in a roundtable discussion (e.g., talk-story circle) format that lasted ~2.5 h. One of the Elder attendees then provided a keynote presentation from her Trail of Tears work with Choctaw women [21,22]. This presentation, entitled “Watering the Seeds of American Indian and Alaska Native Ancestral Love and Wisdom for Native Women’s Health and Well-Being”, was an example of building on the strengths of Native women and Indigenous ways of knowing.

**Figure 3:**
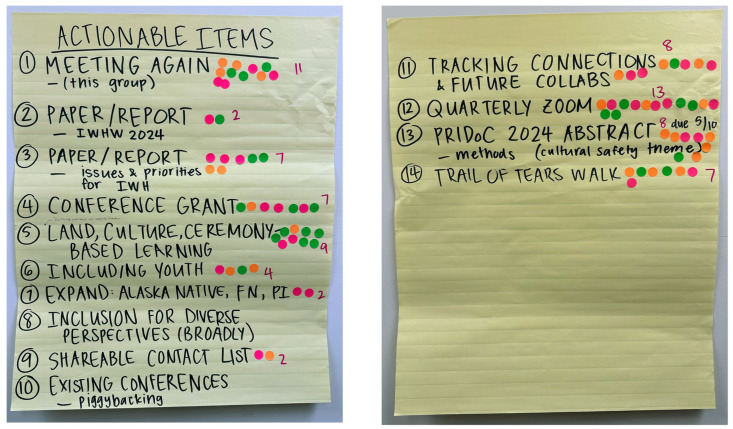
Voting document used after first breakout session on Day 2 to collectively construct action items.

The primary breakout session on Day 2 focused on the question: “Based on what you’ve heard from everyone’s current work and expertise and interests from our talk-story circle earlier today—what are future collaborations, deliverables, next steps that you envision with this group”? Three small groups with 4–5 people in each group spent ~2 h discussing their responses to this query. The groups then recombined for a share-back session; themes were created across the groups, and the full participant group conducted a democratic voting process to determine ‘actionable items’. Visual representations of the results of this voting process can be found in Figure 3 and Table 2.

Each participant completed a short survey after Day 2 to evaluate the workshop. Questions in the survey included a Likert-scale rating of satisfaction with various aspects of the workshop (e.g., small group breakout, voting process) and level of agreement with topics such as diverse representation and feelings of inclusion. There were also three short-answer questions, including “What did you like best about the workshop and why”? and “Please provide suggestions for how we could improve this workshop”. Though only 15 of the participants (out of 23) completed the satisfaction survey, the findings indicate that participants most enjoyed the small group breakout sessions and the peer-to-peer conversation opportunities. One participant shared “I liked hearing about people’s personal stories and perspectives”. Another explained, “in research settings we don’t often get to hear about personal lives and have time to connect on that level, so this [workshop] was meaningful to build trust and understanding”. All participants “agreed or strongly agreed” that the attendees represented diverse backgrounds. Suggestions for improving the workshop included adding a conclusion ceremony, more guest speakers, and more days for the workshop.

## 4. Discussion

The 2024 Indigenous Women’s Health Workshop provided an intentional space to be in community with each other, spend relational time together, and share our stories as Indigenous women and allies who are researchers, practitioners, and students. This workshop provided a safe space for Indigenous women and allies to show up as their full selves (personally and professionally), to center Indigenous ways of knowing, and to bring their values as Indigenous women into discussions and action planning for what they identified as important issues impacting Indigenous women’s health. This workshop highlighted the importance of centering Indigenous voices and creating culturally safe Indigenous environments for dialogue and collaboration. In this discussion, we reflect on how these elements shaped the workshop agenda and format, consider similarities, and discuss what can be learned from global efforts by Indigenous Pacific Islander groups in Aotearoa (New Zealand) and Australia.

### 4.1. Intersectional Approach

The structure of the workshop, guided by intersectionality as a critical framework [16,17], acknowledged the complexity of social determinants of health, settler colonial determinants of health [23], and the overlapping systems of oppression faced by Indigenous women. Attendees represented diverse racial/ethnic identities and professions, including clinical practice, research, and graduate-level students. Many attendees also identified as caregivers, emphasizing the importance of addressing women’s health within their broader roles in families and communities. Intentionally designed to redistribute power dynamics, the discussion sessions facilitated open and authentic sharing, inclusivity, and active participation of individuals who might have felt less comfortable speaking within the larger group. Recognizing the socioeconomic differences among attendees and the normalized gender pay inequities, the provision of stipends, transportation, and meals demonstrated the group’s commitment to reducing barriers to participation and honoring attendees’ time and expertise.

### 4.2. Indigenous Ways of Knowing and Values

Centering Indigenous values, such as relationship building, storytelling, and respect for culture and place, was essential to the workshop’s success. Including cultural protocols set a tone of reverence and connectedness to Native Hawaiian traditions, values, and place, enabling attendees to engage authentically by welcoming everyone to the land and space of the meeting, *oli* (chant), *mele* (song), and *pule* (prayer).

The use of “talk-story” circles, a method of dialogue in Hawai‘i, further embodied Indigenous ways of knowing by sharing personal and professional experiences through storytelling, fostering deep pilina (connection). While this group of women came together because of their professional affiliations, being in community with each other and sharing their stories created a space where they could be their whole selves, which is often not allowed or welcomed in their professional settings. Approaching shared time and space in this way facilitated knowledge exchange and highlighted the relational nature of Indigenous health, emphasizing the interconnectedness of individual, familial, communal, environmental, and spiritual well-being.

### 4.3. Workshop Agenda and Format

Developing the agenda based on co-creation and consensus-building [21] facilitated participant-driven outcomes that reflected diverse perspectives. This approach facilitated the group’s desire to prioritize collective wisdom through sticker voting, iterative group discussions, and collaborative decision-making when identifying the group’s priorities. Further, leaving the workshop with actionable goals and priorities that the group felt were impactful and feasible was achieved through this process, which included plans for future collaboration and expanded representation in subsequent workshops. We developed a professionally designed community-based share-back document to provide a resource for all workshop participants to share the workshop methods and findings with their community and organization [24].

Most importantly, the workshop prioritized an Indigenous holistic view of health, shifting away from disease-focused frameworks in western health approaches. Instead, it focused on strengths-based approaches that include culture, traditional knowledge, and community care. Lifting and centering culture, land, and traditional foods as facilitators of health and well-being further emphasizes the essential role of Indigenous epistemologies [25,26] in developing sustainable health interventions for Indigenous women.

### 4.4. Connections to Global Efforts

The workshop’s approach aligns with efforts by Indigenous people worldwide, particularly the Māori of Aotearoa and the Aboriginal/Torres Strait Islander people of Australia, which have populations of various Pacific Islander groups. Indigenous people in these countries use intersectionality [17], Indigenous ways of knowing [26], and decolonization of research [27] to develop health initiatives that are culturally responsive and sustainable for their communities. For example, Māori health in Aotearoa emphasizes that *whānau* (family), *whakawhanaungatanga* (the process of building relationships), using and reclaiming *tikanga* (cultural protocols and processes), informed by cultural values of *aroha* (compassion and empathy), *manaakitanga* (kindness and hospitality), *mauri* (binding energy), and *wairua* (importance of spiritual well-being), must all be part of health care service delivery for Māori communities [28]. Similar values are present in health care service delivery in Hawai‘i for Native Hawaiians, which were described and emphasized as imperative for holistic, culturally relevant care in the talk-story circles during our workshop.

Future collaborations could involve cross-cultural exchanges with these groups to share best practices and deepen understanding of Indigenous health frameworks, including at the biennial Pacific Regions Indigenous Doctors Congress, where some workshop attendees shared about this gathering. Additionally, incorporating ceremonies, storytelling, and other cultural practices from these regions into future workshops could provide cultural context that parallels with the cultures of other Pacific Indigenous groups, fostering generative discussions around health service delivery for Indigenous communities and solidarity among Indigenous people and allies.

### 4.5. Implications for Future Work

The outcomes and attendees’ experiences of this workshop highlight the need to intentionally create spaces for discussions and collaborative, inclusive creation of solutions to support Indigenous women’s health. Approaches to this must be intersectional, interdisciplinary, and decolonial to ensure Indigenous women’s voices and experiences are centered. Future iterations will consider the following recommendations:

- **Expand Representation:** Incorporate people from underrepresented groups, such as youth, Alaska Native, First Nations, and Pacific Islander communities, including diverse gender identities such as two-spirit *māhū* (Tahiti/Hawaiʻi), *vakasalewa* (Fiji), *palopa* (Papua New Guinea), *faʻafafine* (Sāmoa), *akavaʻine* (Cook Islands), *fakaleiti* (Tonga), *fakafifine* (Niu), and more (MVPFAFF+) [29]. Incorporate more allies working in other disciplines that support Indigenous women’s health (e.g., domestic violence service providers, fertility care providers, social service providers).
- **Power Redistribution:** Redistribution of power is essential to ensuring Indigenous women’s voices, wisdom, expertise, and cultural knowledge are centered. Additionally, hosting gatherings using Native-owned vendors, caterers, and event services redistributes economic power from corporations directly into the community.
- **Foster Global Connections:** Build relationships with Indigenous communities and organizations across the Pacific, including Aotearoa and Australia, to exchange knowledge, as well as other countries with Indigenous communities (e.g., Canada, Taiwan).
- **Deepen Cultural Understanding:** Create opportunities for cultural, ceremonial, and land-based learning and healing, such as those inspired by walking the Trail of Tears [30,31,32], planting *kalo* (taro) in a *loʻi* (taro patch), or restoring *loko iʻa* (fish ponds).
- **Sustain Momentum:** Organize a regular meeting schedule and collaborative projects to maintain and deepen relationships among the group to advance Indigenous women’s health.

## 5. Conclusions

Intentional gatherings created to hold Indigenous women and Indigenous knowledge systems at the center while fostering intersectional and interdisciplinary approaches are essential to identifying Indigenous women’s health priorities. The development and facilitation of this workshop offer a way forward for advancing health equity and honoring Indigenous women’s holistic well-being.

## Figures and Tables

**Table 1:** Key health priorities, facilitators, and challenges for Indigenous women’s health as generated from consensus building process on Day 1.

Key Health Priority	Facilitators to Achieving Key Health Priorities	Challenges to Achieving Key Health Priorities
Cancer Cardiovascular and metabolic disease Healthy lifestyles Maternal health Mental health Reproductive health Sexually transmitted infections (STIs) Substance use	Agency, self-advocacy Connections (to healthcare, resources, support systems) Culture, land, traditional foods Intergenerational, multigenerational	Access to data Access to healthcare, provider shortages, rural areas Patriarchy Racism and microaggressions Representation Violence and historical cultural trauma Wages, cost of living

**Table 2:** Action items from Indigenous Women’s Health Workshop participants as generated from consensus building process on Day 2.

Action Items for the Indigenous Women’s Health Workshop Participants
Continue meeting as a group via quarterly teleconference (e.g., Zoom) Focus on land, culture, and ceremony-based learning (e.g., consider Trail of Tears * walk for next workshop) Piggyback future Indigenous Women’s Health Workshops on other related conferences Provide shareable contact list for all participants in this workshop Seek conference grant to support meeting again in ~1 year Submit abstract(s) to 2024 Pacific Region Indigenous Doctors Congress (PRIDoc) conference To enhance diverse perspectives, expand participation in this workshop to youth, Alaska Native, First Nations, and Pacific Islander representation (among others) Track collaborations and connections between participant workshops Write publishable paper/report from this workshop (topics: methods of conference and/or key topics of focus for Indigenous women’s health)

* The Trail of Tears, part of the Indian Removal Act of 1830, was a forced relocation of Indigenous peoples from their homelands in the Southeastern United States to Indian Territory (present day Oklahoma) [9].

## Data Availability

Data are contained within the article.
